# Pneumomediastinum following Endobronchial Ultrasound– Guided Transbronchial Needle Aspiration: A Case Report

**Published:** 2017

**Authors:** Ines Maria Grazia Piroddi, Piergiorgio Gatto, Alessandro Perazzo, Cornelius Barlascini, Antonello Nicolini

**Affiliations:** 1 Respiratory Diseases Unit, Hospital of Sestri Levante, Italy; 2 Hygiene and Health Medicine Unit, Hospital of Sestri Levante, Italy

**Keywords:** Endobronchial ultrasound-guided needle aspiration, Mediastinal adenopathy, Pneumomediastinum, Non-Hodgkin’s lymphoma

## Abstract

Endobronchial ultrasound-guided transbronchial needle aspiration (EBUS-TBNA) is an effective and safe technique associated with a very low complication rate for the sampling of lymph nodes in those presenting with mediastinal and hilar adenopathy.” We report a rare case describing the development of pneumomediastinum following EBUS-TBNA in a young patient with mediastinal lymphadenopathy secondary to non-Hodgkin’s lymphoma. Conservative treatment led to spontaneous resolution of the pneumomediastinum. Pneumomediastinum is a rare but possible complication of EBUS-TBNA. Careful follow-up can reduce its severity and the associated morbidity.

## INTRODUCTION

Endobronchial ultrasound-guided transbronchial needle aspiration (EBUS-TBNA) is an effective technique for the sampling of mediastinal and hilar adenopathy (1,2). Moreover, EBUS-TBNA is a safe procedure associated with a very low complication rate (3). A meta-analysis reported a complication rate of 0.15% (1). In a registry study that evaluated 1,317 patients, 1.44% of the patients demonstrated complications (3 developed bleeding requiring intervention, 7 developed pneumothorax, 4 developed sustained hypoxia, 3 developed respiratory failure within 24 hours, 1 developed clinically significant airway injury, and 1 developed post procedural hypotension) (4).

In a nationwide survey performed by the Japan Society for Respiratory Endoscopy, pneumothorax was documented in 2 patients (0.03%), 1 of whom required tube drainage (1). Pneumomediastinum is an extremely rare complication of EBUS-TBNA, and only a few sporadic cases have been described in the literature (5–8). We report a patient who developed pneumomediastinum following EBUS-TBNA of mediastinal lymph node stations.

## CASE SUMMARIES

**Figure 1.**
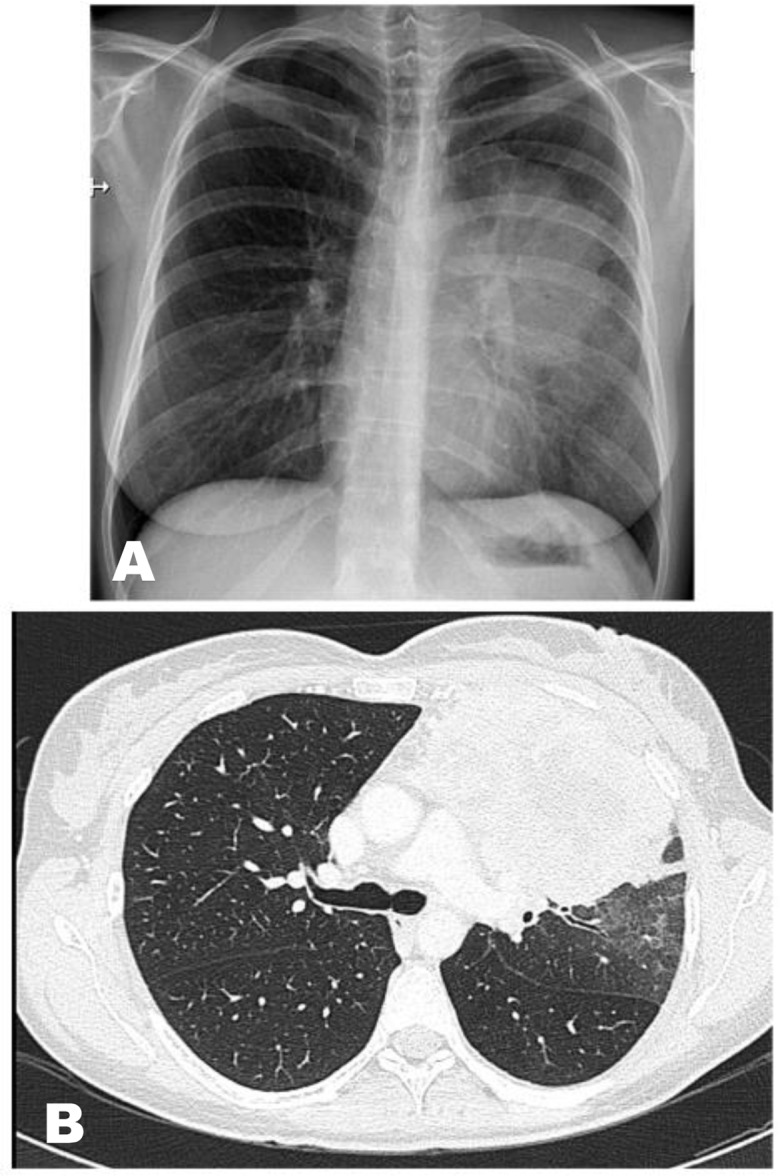
A) Chest X ray: left sided perihilar opacity, B) Computed tomography of thorax: left upper lobe solid mass

**Figure 2.**
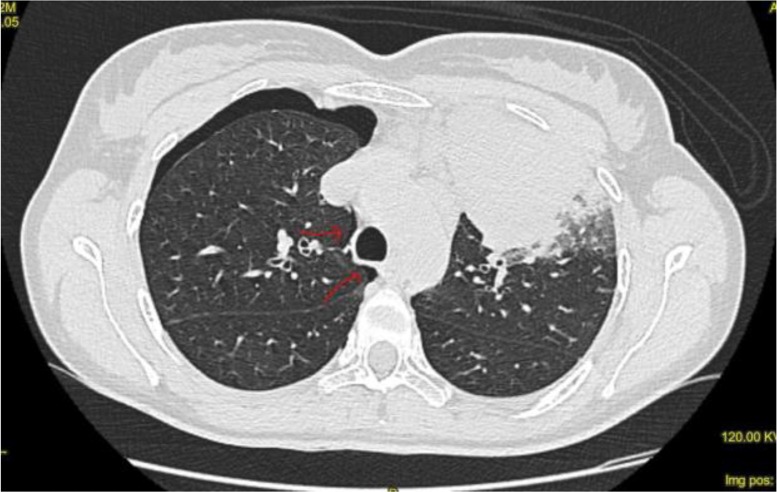
Computed tomography of thorax: pneumothorax involving the right side and slight pneumomediastinum (shown by the arrows)

A 42-year-old woman presented to the outpatient Pneumology Clinic with a complaint of a persistent cough without fever over a month and the onset of chest pain a week prior to presentation. A chest X-ray revealed a left-sided perihilar opacity (Figure 1A). She reported a negative medical history and was prescribed the antibiotic levofloxacin. Computerized tomography (CT) of the thorax demonstrated a solid mass (10×12 cm in diameter) in the left upper lobe, as well as enlarged superior mediastinal and aorto-pulmonary lymph nodes (Figure 1B). Positron emission tomography demonstrated pulmonary neoplasm with a standardized uptake value of 31 and mediastinal lymphadenopathy. Fibrobronchoscopy and EBUS-TBNA were performed in the outpatient clinic, aimed at diagnosing and staging the probable lung tumor. Using a 21-gauge needle, she underwent a bronchoscopy with EBUS-TBNA of subcarinal lymph node stations (nodal station 7) and suspicious histopathological tissue at the upper left bronchus. The procedure was performed under deep sedation using midazolam and propofol; however, resistant cough was noted. Upon completion of the procedure, she complained of acute chest pain, which regressed immediately, and a physical examination was negative following which she returned home. Cytological results of all biopsies obtained with EBUS-TBNA were negative. CT-guided fine-needle aspiration (FNA) performed 3 days later revealed a right-sided pneumothorax associated with a small pneumomediastinum (Figure 2). The patient was admitted to the Department of Internal Medicine for 4 days of observation and responded to conservative management. Based on extranodal dissemination, the cytological samples obtained via FNA that were positive for non-Hodgkin’s diffuse large B-cell lymphoma, were later classified (staged) as samples showing a stage IV tumor. We obtained the patient’s consent to publish this case report.

## DISCUSSION

The clinical presentation of pneumomediastinum can occasionally be misdiagnosed or completely missed because of its vague symptoms (9–11). Most patients demonstrate typical symptoms (Table 1), although good clinical discernment supported by objective evidence is necessary to establish a conclusive diagnosis (10). Chest pain and dyspnea have been reported as the predominant symptoms followed by cervical pain, cough, and dysphagia (10,11). Coughing has been considered a precipitating factor in approximately 50% of patients (9). We could conclude that in our patient, her persistent cough contributed to the development of pneumomediastinum. Reportedly, cough may serve as an important precipitating factor associated with this complication (5,6, 9). A chest X-ray may be normal in approximately 30% of the patients; therefore, thoracic CT is considered the most sensitive diagnostic modality (11,12).

**Table 1.** Pneumomediastinum most symptoms (9,11)

Chest pain	37.2%
Cervical pain	17.9%
Dyspnea	9.6%
Cough	7.7%
Crepitus sensation	6.4%
Dysphagia	4.5%
Abdominal pain	1.9%
Pharyngeal enlargement	1.3%

Bed rest and conservative management such as the prophylactic use of analgesics and antibiotics in addition to limitation of oral intake are indicated in patients presenting with pneumomediastinum (10,12).

Management of pneumomediastinum and pneumopericardium depends upon the clinical severity and underlying etiology of these conditions. Most patients respond adequately to the conservative management strategy outlined above. In rare instances, surgery may be required to treat pneumomediastinum that occurs secondary to severe tracheobronchial disruption (5). Complications of EBUS-TBNA are similar to those observed with conventional TBNA—primarily, pneumomediastinum, mediastinitis, bleeding, and other rare complications. Pneumothorax, infection, and airway alterations have also been reported in rare instances (6).

## CONCLUSION

We have described a rare complication of EBUS-TBNA. Our patient was known to experience chest pain prior to the bronchoscopy; however, the sudden regression in her chest pain noted after the bronchoscopy led to an underestimation of the possible procedural complication, although the patient’s pneumomediastinum resolved with supportive care. Although rare, clinicians must remain mindful of the complications associated with EBUS-TBNA, and careful follow-up is warranted to reduce the associated morbidity.
